# Be Health*e* for Your Heart: A Pilot Randomized Controlled Trial Evaluating a Web-Based Behavioral Intervention to Improve the Cardiovascular Health of Women with a History of Preeclampsia

**DOI:** 10.3390/ijerph17165779

**Published:** 2020-08-10

**Authors:** Melinda J. Hutchesson, Rachael Taylor, Vanessa A. Shrewsbury, Lisa Vincze, Linda E. Campbell, Robin Callister, Felicity Park, Tracy L. Schumacher, Clare E. Collins

**Affiliations:** 1 Priority Research Centre for Physical Activity and Nutrition, School of Health Sciences, Faculty of Health and Medicine, The University of Newcastle, Callaghan, NSW 2308, Australia; rachael.taylor@newcastle.edu.au (R.T.); vanessa.shrewsbury@newcastle.edu.au (V.A.S.); tracy.schumacher@newcastle.edu.au (T.L.S.); clare.collins@newcastle.edu.au (C.E.C.); 2 School of Allied Health Sciences & Menzies Health Institute Queensland, Griffith University, Gold Coast, QLD 4222, Australia; l.vincze@griffith.edu.au; 3 School of Psychology, Faculty of Science, The University of Newcastle, Callaghan, NSW 2308, Australia; linda.e.campbell@newcastle.edu.au; 4 Priority Research Centre for Physical Activity and Nutrition, School of Biomedical Sciences and Pharmacy, Faculty of Health and Medicine, The University of Newcastle, Callaghan, NSW 2308, Australia; robin.callister@newcastle.edu.au; 5 Department of Maternal Foetal Medicine, John Hunter Hospital, Newcastle, NSW 2305, Australia; Felicity.Park@hnehealth.nsw.gov.au; 6 Priority Research Centre for Health Behaviours, Department of Rural Health, Faculty of Health and Medicine, University of Newcastle, Tamworth, NSW 2340, Australia

**Keywords:** cardiovascular disease, preeclampsia, postpartum, health behavior, women, prevention, eHealth

## Abstract

This pilot randomized controlled trial (RCT) aimed to determine the acceptability and preliminary efficacy of a web-based cardiovascular disease (CVD) prevention intervention for women following preeclampsia. Australian women with a recent history (≤4 years post diagnosis) of preeclampsia were randomized into two study arms: (1) Be Health*e* for your Heart, a web-based behavioral intervention or; (2) Control, access to the National Heart Foundation website. Assessments were conducted at baseline, and after three months. Intervention acceptability and impact on absolute CVD 30-year risk score, CVD risk markers and health behaviors were assessed. Twenty-four of 31 (77.4%) women completed the three-month assessment. Eleven out of 13 intervention participants (84.6%) agreed/strongly agreed they were satisfied with the program, with a mean score of 4.2 ± 0.9 (maximum of five). There were no significant between or within group differences in absolute CVD risk, CVD risk markers or health behaviors from baseline to three months. Women with a history of preeclampsia were successfully recruited and retained and they reported high levels of acceptability with the Be Health*e* for your Heart program. Further research is therefore needed from powered trials to determine the impact of web-based lifestyle interventions on CVD risk in this at-risk group.

## 1. Introduction

Cardiovascular disease (CVD) is the leading cause of death in women, contributing to 35% of female deaths worldwide [1]. Some CVD risk factors are unique to women, including the incidence of hypertensive disorders of pregnancy (HDP) such as preeclampsia and gestational hypertension. Worldwide, HDP affects 5–10% of pregnancies and has increased by 25% in the past decade [2,3]. As the causes [4] and early diagnosis of HDP [5], which may lead to prevention of CVD, are yet to be established, a focus on preventing the long-term consequences of HDP is required. A recent meta-analysis of 22 studies (18 cohort and four cross-sectional studies) including more than 6.4 million women (>258,000 with a history of preeclampsia) indicated that preeclampsia is an independent predictor for future risk of heart failure [Relative Risk (RR): 4.19, 95% Confidence Interval (CI): 2.09–8.38], coronary heart disease (RR: 2.50, 95% CI: 1.43–4.37), CVD mortality (RR: 2.21, 95% CI 1.83–8.26), and stroke (RR: 1.81, 95% CI: 1.29–2.55) [6]. Findings from three systematic reviews also provide supporting evidence that women with a history of preeclampsia have an increased future risk of developing CVD [7,8,9]. Notably, the highest incidences of CVD morbidity and mortality are reported in the first ten years after HDP [10,11]. Furthermore, women who experience recurrent preeclampsia in a subsequent pregnancy may be more susceptible to developing CVD compared to women with a single exposure of preeclampsia [12]. 

Internationally, clinical guidelines acknowledge that HDP is a risk factor for CVD and recommend routine assessment and monitoring for the management of cardiovascular health in women [13]. The International Society for the Study of Hypertension in Pregnancy (ISSHP) recommends educating women with a history of HDP about their future CVD risk and modifiable risk factors (i.e., excess body weight, physical inactivity, poor diet and smoking), as well as regular monitoring of blood pressure, fasting lipids and blood glucose levels [13]. Similar recommendations are provided by The Society of Obstetric Medicine of Australia and New Zealand (SOMANZ) Guideline for the Management of HDP [14]. Despite these recommendations, review findings indicate that healthcare providers and women have limited or no knowledge of the association between HDP and CVD [15]. 

A 2019 systematic review of postpartum interventions to reduce CVD risk in women after HDP identified only two published randomized controlled trials (RCTs) evaluating such interventions [16]. Findings from this review highlight that sufficiently powered RCTs are urgently required to determine interventions that are effective for lowering the risk of CVD after HDP. 

Previous studies have reported that there are significant barriers for reaching, engaging and retaining postpartum women in lifestyle-based interventions, including a lack of time due to infant/family care, low energy levels and changes in personal priorities [17,18]. Evidence indicates that delivering lifestyle-based interventions via information and communication technologies, including Electronic Health (*e*Health) and Mobile Health (*m*Health), may alleviate these barriers experienced by postpartum women [19,20].

Therefore, the aim of this pilot RCT was to determine acceptability and preliminary efficacy of a web-based lifestyle behavioral intervention (Be Health*e* for your Heart) targeting women with a recent history of preeclampsia. The study: (1) evaluated intervention acceptability (satisfaction, usability, appropriateness, usage), and (2) estimated pre-to-post intervention impact on absolute CVD 30-year risk score, CVD risk markers (body fat percentage, body mass index (BMI), waist circumference, blood pressure, blood lipids and glucose), health behavior risk factors (dietary intake, physical activity and stress) and general health and wellbeing, compared with the control group.

## 2. Materials and Methods

### 2.1. Trial Design

A three-month, two-arm parallel group pilot RCT was undertaken at The University of Newcastle (UON), New South Wales (NSW), Australia. Participants were randomly allocated to Be Health*e* for your Heart or Control, with assessments conducted at baseline and after three months. The study was prospectively registered with the Australian New Zealand Clinical Trials Registry (ANZCTR): 12618001528246, and the study protocol has been published [21].

### 2.2. Participants

Women aged 18 to 45 years with a recent history (within four years of diagnosis) of preeclampsia were recruited from November 2018 to March 2019. Detailed inclusion and exclusion criteria are described in Table 1. Participants were recruited simultaneously using multiple strategies: (1) all women who were treated at John Hunter Hospital (JHH), NSW, Australia for preeclampsia within the last four years were mailed information about the study; (2) the Australian Action for Preeclampsia organization advertised the study on their social media accounts and online newsletter; (3) general practitioners (GPs) and other services that have contact with women within four years of birth, such as childcare centers, playgroups, child recreation activities and community centers, were provided with recruitment materials (posters, flyers, social media posts) to share with potentially eligible women; and (4) women with a history of preeclampsia who completed a previous survey [22] conducted by the research team and agreed to be recontacted were emailed an invitation to participate.

The pilot RCT obtained ethics approval from the Hunter New England Human Research Ethics Committee (18/09/19/4.09) and was registered with The UON Human Research Ethics Committee. The trial was undertaken in compliance with the Declaration of Helsinki [23]. Interested participants accessed an online participant information statement and survey to assess their study eligibility. Eligible participants were provided a consent form, and all provided written informed consent prior to study commencement.

### 2.3. Intervention

Participants allocated to the intervention group were provided access to a three-month lifestyle behavior intervention (Be Health*e* for your Heart) delivered via the program website and weekly email newsletters. The program focused on modifiable CVD risk factors (nutrition, physical activity, stress management and weight management), which were promoted through 14 evidence-based program recommendations. Program recommendations focused on eating plenty of fruit, vegetables and wholegrain cereals, eating a variety of healthy protein sources, choosing reduced-fat dairy, selecting healthy unsaturated fat choices, and limiting salt intake through the use of herbs and spices [24], gradually building up to 2.5 h of moderate intensity physical activity or 1.25 h of vigorous intensity physical activity each week, doing muscle strengthening activities at least two days each week, limiting the amount of time spent in prolonged sitting and breaking up sitting time [25], identifying and managing emotional stress, returning to pre-pregnancy weight, and reaching and maintaining a healthy weight [26]. Participants were provided with access to five program components, which were aligned with 21 different behavior change techniques [27]. The program components were:

- *How Healthy is your Heart?* A brief survey was available on the website from enrolment to evaluate each participant’s current eating habits, physical activity, stress levels and body weight. Automated individualized feedback was provided based on participant’s responses comparing current behaviors to the program recommendations.
- *My goals:* Allowed participants to select up to four behavior change goals consistent with the program recommendations, and to record strategies for achieving those goals. This component was available on the website throughout the three months.
- *Track my progress:* Allowed participants to self-monitor their progress by answering a series of questions related to their goals. Feedback was provided on their progress towards achieving their goals and the program recommendations. This component could only be completed once the *My goals* component was completed.
- *Resources:* Comprehensive written information related to the program recommendations was provided. All resources were available throughout the three months.
- *Email newsletters:* Participants were sent a weekly newsletter, which focused on a different program recommendation each week, and prompted participants to use the website components.

### 2.4. Control Group

Participants allocated to the control group were sent an email with links to the National Heart Foundation of Australia website (www.heartfoundation.org.au). They were provided with access to the Be Health*e* for your Heart intervention after completion of the three month follow-up appointment.

### 2.5. Outcome Measures 

#### 2.5.1. Acceptability (Primary Outcome)

Intervention participants completed a 40-item online process evaluation survey via Qualtrics (Qualtrics, Seattle, Washington, US) after the three-month intervention. They were asked to rank statements related to program acceptability (five-point Likert scale, Strongly agree (=5) to Strongly disagree (=1)). They were asked to indicate whether they used each of the five program components, and if they reported using the component, they were asked to rank (five-point Likert scale, Strongly agree (=5) to Strongly disagree (=1)) the attractiveness (*“was visually appealing”*), comprehension (*“provided me with useful information about”*), usability (*“was easy to use/receive”*) and ability to persuade/engage *(“helped me to attain my weight loss goals”, “motivated me” and “made me feel accountable”*) aspects of the components, as well as answer two open questions to report what they liked or disliked about the component. If they reported they did not use the component, they were asked an open question to report why they did not use it. In addition, each participant’s use of the intervention components was objectively tracked, including whether *How Healthy is your Heart, My goals and Track my progress* were completed, as well as whether email newsletters were opened. 

#### 2.5.2. Preliminary Efficacy (Secondary Outcome)

All secondary outcomes were measured at baseline and after three months to allow evaluation of change in outcomes during the intervention period. Participant completion of each outcome measure was also tracked, as a measure of the feasibility of the data collection procedures. Secondary outcomes, including impact on absolute CVD risk, CVD risk markers and health behaviors, are described in Table 2. 

#### 2.5.3. Other Measures

At baseline participants answered questions about socio-demographic characteristics (e.g., age, country of origin, language spoken at home, highest level of education, individual and household income, marital status, working status, and living situation/family structure), as well as optional questions about their pregnancy history (number of pregnancies, their outcome and whether they were complicated by preeclampsia or other pregnancy complications). 

### 2.6. Sample Size

A powered sample is not required for a pilot study [36], so a maximum target of 90 participants (45 per group) was set a priori, as this was anticipated to be feasible within the funding timeline and budget.

### 2.7. Randomisation and Blinding

The randomization sequence was generated by an independent statistician, using a random number function in Microsoft Excel. A randomized block design, with a block size of six, and stratification by time since last pregnancy complicated by preeclampsia (3 months to <1 year, ≥1 to <2 years, ≥2 to 4 years) was used. A concealed envelope was provided to all participants after completion of baseline measurements to reveal the study condition assigned. Researchers involved in the collection of physical measurements were blinded to participant group allocation. Participant blinding was not possible due to the nature of the intervention and control conditions. 

### 2.8. Statistical Methods

All analyses were performed using Stata/IC Version 16 (Stata, College Station, TX, USA). Data are presented as mean ± standard deviation (SD) or median, interquartile range (IQR) for continuous variables, and counts (percentages) for categorical variables. Changes in the impact on absolute CVD risk score, CVD risk markers and health behaviors were determined, and differences between groups examined. Analyses for the preliminary efficacy outcomes were conducted on an intention-to-treat basis (all participants who were randomized to groups and completed baseline assessments) and for completers only (those who provided data at three months). The effect of treatment on the efficacy outcomes were assessed using linear mixed models. Each efficacy outcome was an outcome in a model, with time (baseline, three months) and treatment group (intervention, control) as predictors, and group × time as an interaction term. The *p*-value of the interaction term was used to determine the statistical significance of any difference between treatment groups in the change from baseline. Effect sizes were calculated using Cohen’s *d* (M_1 change score_− M_2 change score_)/SD_pooled (change scores)_. Intervention acceptability is presented as the mean ± SD, with higher scores (maximum of five) indicating greater acceptability. For qualitative data analyses, answers from open questions were categorized into themes. 

## 3. Results

### 3.1. Recruitment

**Figure 1:**
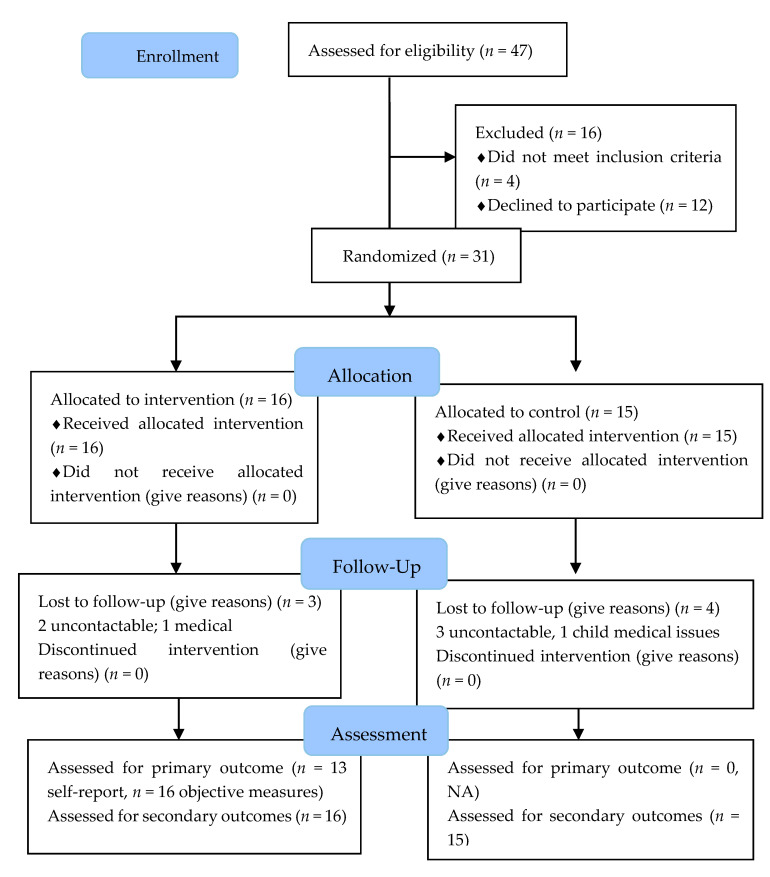
The flow of participants in Be Health*e* for Your Heart study.

Participant flow is described in Figure 1. Over the five-month recruitment period, 47 individuals expressed interest in participating in the study and completed the online eligibility questionnaire. Of those screened for eligibility, 15 reported finding out about the study from the letter received from JHH, 13 from the Australian Action for Preeclampsia advertisements, and one responded to the email invitation due to completing our previous survey. A further 15 participants indicated they found out via social media or the media generally, and therefore likely due to services that have contact with women within four years of birth. Two participants reported they found out about the study from a friend or family member. Notably, no participants reported they were informed about the study by their GP.

From the online eligibility questionnaire, 45 individuals were deemed eligible for the study, of whom 38 consented to participate, and 31 attended baseline assessments and were randomized to the intervention (*n* = 16) or control (*n* = 15) groups. Of the 14 eligible participants who did not proceed with the study, five were unable to be contacted, three reported new health issues that prevented participation and five reported they could not attend baseline assessments, citing work commitments, cost of travel, and carer responsibilities for non-attendance. Two were confirmed as not meeting eligibility criteria (>4 years post-preeclampsia, unable to attend follow-up appointment as moving interstate).

### 3.2. Participant Characteristics

The characteristics of participants at baseline are described in Table 3. Briefly, participants had a mean ± SD age of 33.4 ± 4.6 years, most were born in Australia (93.6%) and were married (80.7%). Most participants (80.6%) had one pregnancy complicated by preeclampsia. For their most recent pregnancy complicated by preeclampsia, many (60%) were diagnosed at <34 weeks gestation, 10% at 34–37 weeks, 20% at ≥37 weeks, and 10% were diagnosed with preeclampsia postpartum. Many participants (67.7%) were ≥2 to 4 years since their pregnancy complicated by preeclampsia, while 22.6% were 3 months to <1 year postpartum, and 9.7% ≥1 to <2 years.

In terms of CVD risk factors at baseline, 83.9% of the participants were classified as having overweight or obesity (BMI ≥ 25 kg/m^2^) and 77.8% had a waist circumference >80 cm. Two participants’ blood pressure placed them in the high-normal range (i.e., systolic blood pressure (SBP) 130–139 and/or diastolic blood pressure (DBP) 85–89), two participants’ blood pressure was consistent with Grade one hypertension (SBP 140–159 and/or DBP 90–99), and one participant’s blood pressure was consistent with Grade two hypertension (SBP > 160 and/or DBP > 100). Four participants had fasting total cholesterol >5.5 mmol/L, five participants had LDL-cholesterol >3.5 mmol/L, 11 participants had HDL-cholesterol <1.3 mmol/L and four participants had triglycerides >2.0 mmol/L. There was no evidence of impaired fasting glucose, impaired glucose tolerance or Type II diabetes with all participants fasting blood glucose <6.1 mmol/L. Six participants were classified as intermediate risk of CVD based on the Framingham 30-year Risk Score (Hard CVD), with no participants classified as high risk.

### 3.3. Participant Retention

Twenty-four of 31 participants (77.4%) completed the three-month assessments. There was no significant difference in retention rates between the intervention (*n* = 13, 81.3%) and control (*n* = 11, 73.3%) groups. One participant withdrew due to personal medical reasons, and another due to acute medical needs of her child. The other five dropouts could not be contacted.

### 3.4. Acceptability

Thirteen intervention participants completed the process evaluation survey. Eleven participants (84.6%) agreed/strongly agreed they were satisfied with the program, with a mean score of 4.2 ± 0.9 out of a maximum of five (strongly agree). The same proportion (84.6%) agreed/strongly agreed they would recommend the program to other women with a history of preeclampsia (mean score: 4.5 ± 0.7), that it met their expectations (4.5 ± 0.7) and the program recommendations were relevant to women with a history of preeclampsia (4.2 ± 0.7). Ten (76.9%) agreed/strongly agreed the program was clearly designed for women with a history of preeclampsia, and twelve (92.3%) agreed/strongly agreed the program was appropriate as it was delivered using technology (4.4 ± 0.6).

For the five program components, the highest proportion of participants indicated they read the website resources (*n* = 12, 92.3%), completed the *How Healthy is your Heart?* tool (*n* = 11, 84.6%), and read the email newsletters (*n* = 10, 76.9%). A lower number of participants indicated they used the *My goals* (*n* = 7, 53.8%) and *Track my progress* (*n* = 4, 30.8%) tools. The two participants who did not complete *How Healthy is your Heart?* indicated they were unaware it was available *(“I read the info but didn’t even realise I hadn’t completed the tool”).* Of the six participants who did not complete *My goals*, the predominant reason was lack of time (*“I was busy when I first looked at it and then I forgot”).* Of the nine participants who did not use the *Track my progress* tool, the main reasons given were lack of time (*“Mostly time constraint”*), as well as uncertainty of the usefulness of the tool (*“I felt like I wasn’t able to achieve my goals in the timeline I wanted and didn’t think there was much to track”* ). No reasons for non-engagement were provided by those who reported they did not read the newsletters or website resources. 

Table 4 summarizes the mean rankings for program acceptability for the five program components. Mean satisfaction ranged from 3.6–4.2. The email newsletters (4.2 ± 0.7) and the *How Healthy is your Heart?* (4.2 ± 0.4) were the program components participants were most satisfied with whereas the lowest level of satisfaction was with *Track my progress* (3.6 ± 0.8). All program components scored highly for attractiveness *(“was visually appealing”,* mean 4.1–4.4) and usability (*“Was easy to access/use,* mean 4.0–4.3). Comprehension (“*Provided me with useful information about…*”) also scored highly for each program component, with all but one component having a mean score > 4. The program components’ ability to persuade/engage participants (*“Helped me attain my goals”, “Made me feel accountable”, “Motivated me”*) scored more variably (range 3.4–4.3), with the *Track my progress* tool generally scoring higher in these categories (range 4–4.3) for those who used it.

Responses to the open question that asked what participants liked most about each program component indicated *How Healthy is your Heart?* was easy to use and understand (“*I liked that it wasn’t complicated and looking at your score as colored hearts was clever. I was able to see exactly where I needed work and where I was already doing well.”*). Participants reported they found the website resources informative and they provided a good overview of the different program recommendations *(“They were easy to read and informative without being an information overload”*). Similarly, participants reported the email newsletters were easy to understand and informative, and also found them a good weekly reminder (*“It was a weekly reminder to check in on how I was going with my goals. And the information was helpful along the way too”*). For the goal setting tool, the participants commented on the ease of use, along with the fact they were encouraged to set realistic goals *(“They were small realistic goals”).*

Responses to the open questions asking what could be improved for each program component were diverse, with no two respondents providing the same suggestion. Responses included improving usability of the existing tools (e.g., adding a status bar to the *How Healthy is your Heart*? tool to indicate progress, making it easier to change and set new goals), changing the delivery mode of different components (e.g., making *How Healthy is your Heart?* available as an app, providing more information in the email newsletters in place of the website resources), and adding new program features (e.g., addition of text message reminders to complete the *Track my Progress* tool). One respondent wanted an existing feature removed, that is, in the *Track my Progress* tool where participants reported progress towards their goal and received feedback compared to the goal they set, as well as the program recommendations (*“I did not like that I was being compared to a goal I did not set, it made me feel like the progress I had made was not good enough”*).

Objective data indicated that nine intervention participants (56.3%) completed *How Healthy is your Heart?* and two participants started it but did not complete it. Four participants (25%) completed the *My goals* and the *Track my Progress* tools. All intervention participants opened at least two email newsletters (Mean 9.5, Range 2–13). The highest open rates were in weeks one and two when 15 participants opened the newsletters (93.8%). The lowest open rate was in week 11 when 8 (50%) opened the newsletter. 

### 3.5. Preliminary Efficacy

The majority of outcome measures were obtained from all participants who completed baseline and three-month appointments. A blood sample was unable to be drawn for one participant at baseline, therefore preventing the calculation of a Framingham 30-year CVD risk score. Implausibly high data were reported for the IPAQ for seven participants at baseline and six participants at three months. Table 5 describes the preliminary efficacy outcomes. 

No between group differences in the change in preliminary efficacy outcomes were detected from baseline to three months. No within group changes in outcomes were detected from baseline to three months in the intervention group. In the control group there was a significant increase in total cholesterol (mean change: 0.54 mmol/L, 95% CI: 0.04, 1.04) from baseline to three months but no other significant changes. 

## 4. Discussion

This pilot RCT determined the acceptability and preliminary efficacy of a web-based lifestyle behavioral intervention targeted to women with a recent history of preeclampsia (Be Health*e* for your Heart) for the prevention of CVD. Overall the acceptability (satisfaction, usability, appropriateness and usage) of Be Health*e* for your Heart from the participant’s perspective was high, however acceptability was inconsistent across the five program components. No statistically significant differences were detected between the Be Health*e* for your Heart and control group’s changes from baseline to three months for absolute CVD risk, CVD risk markers or health behaviors. 

### 4.1. Participant Recruitment and Retention

Over a five-month period the study recruited 31 women with preeclampsia in the past four years from the Greater Newcastle region of NSW, Australia. There are approximately 5100 births within the region each year, and 1.4% of pregnancies in NSW are complicated by preeclampsia [37]. Therefore, approximately 286 women were potentially eligible to participate in the study, meaning 11% of the intended target group was recruited. A variety of recruitment strategies were utilized within the study, with the letter sent from the main birthing hospital in the region, and advertisements via social media, particularly by the Australian Action for Preeclampsia organization, contributing to the most study enquiries. Due to the passive nature of the recruitment strategies it is not possible to determine how many women within the target group were aware of the study. Notably, the study achieved a recruitment rate of 66%, with 31 of the 47 women who expressed interest participating. There were low numbers of interested participants who were deemed ineligible, suggesting the recruitment materials and participant information statement adequately informed participants about the study requirements. There were higher numbers of interested and eligible participants who elected not to proceed with the study or were lost to follow-up, with indications they faced barriers to study participation, particularly in relation to attending the face-to-face data collection appointments. Comparatively, the one other published RCT to have evaluated a CVD lifestyle prevention intervention for women following preeclampsia recruited 151 women with preeclampsia within the past five years over an 11-month period from across the United States using websites and social media. They had 1493 women express interest in participating, most of whom were deemed ineligible (*n* = 871), while others declined participation (*n* = 43), were lost to follow-up (*n* = 167) or did not complete the recruitment process as the target sample size for the study was achieved (*n* = 260) [38]. Overall, the findings from the RCT provide support for the recruitment materials and strategies utilized in the study. However, the RCT highlights that if a larger powered efficacy study were to be undertaken to evaluate the Be Health*e* for your Heart intervention a longer timeframe for recruitment would be required and/or a larger geographical region for recruitment utilized.

Previous research has highlighted the challenges of retaining women in postpartum lifestyle interventions. The current study achieved a reasonable retention rate of 77.4% at three months, which is lower than the one previous similar trial (>90% in both intervention and control group after nine months) but within the range of retention rates reported in systematic reviews of postpartum lifestyle interventions [39,40]. In the current study, only minimal strategies were used to support participant retention, including provision of reimbursement for completion of data collection (AU$20 baseline and AU$40 at follow-up) and multiple reminders via email, phone and text message to book-in for follow-up data collection appointment and attend upcoming appointment. A notable difference between the current study and the one previous similar RCT from the USA was participants in that trial did not need to attend in-person data collection appointments, with all data collection completed online, with key equipment required for data collection (i.e., blood pressure, weight) provided to each participant. Such an approach to study design has the potential to overcome key challenges to study participation faced by postpartum women (e.g., time constraints due to carer responsibilities), and may positively impact both recruitment and recruitment rates [41].

### 4.2. Intervention Acceptability

Overall, the acceptability of the Be Health*e* for your Heart was high. Many participants self-reported high levels of satisfaction with the intervention, engaged with the five program components, and rated them consistently high for usability and appropriateness. However, the usability, appropriateness and usage of the five program components varied, suggesting some program aspects were more acceptable to participants than others. The most positive findings were for the email newsletters, with high levels of user engagement based on overall email open rates (Mean 9.5/13) and participant reported usability and appropriateness. Both the *How Healthy is your Heart?* tool and the website resources received positive feedback in terms of usability and appropriateness, but based on participant usage data, engagement with these components was moderate. Usage of the *My goals* and *Track my Progress* components was lower, and overall satisfaction was also lower for these components compared to the others, although still overall reasonable. Both the *My goals* and *Track my Progress* components provide key behavior change techniques (e.g., goal setting, action planning, self-monitoring, feedback) and are likely imperative to successful behavior change [27]. The qualitative responses from intervention participants suggest that to improve engagement with these program components greater consideration must be given to how to promote the usefulness and importance of the components, as well as overcoming the perceived time commitment required for completion. For example, small changes to how the components are presented and described on the website could be made (e.g., highlighting why goal setting is important, and how much time each component will take to complete). Alternatively, greater support may be required for participants to complete these key program components (e.g., initial consultation with a health professional either in-person or online to guide completion). Such minor and major alterations to the Be Health*e* for your Heart intervention could be explored in future studies or inform the design of new approaches to CVD prevention for women following preeclampsia. 

### 4.3. Preliminary Efficacy

The study was not powered to detect between group differences in outcomes, and therefore no statistically significant differences were detected between the Be Health*e* for your Heart and control groups for change from baseline to three months in absolute CVD risk, CVD risk markers or health behaviors. There were low effect sizes for the majority of outcomes, signifying trivial differences between the intervention and control groups. However, there were moderate effect sizes for total cholesterol (−0.58), LDL-C (−0.56), polyunsaturated fatty acid intake (0.51), sodium intake (−0.62) and resistance-based exercise (−0.75) suggesting potential for positive impacts of the intervention on health behaviors and CVD biomarkers. In one similar trial conducted to date, Rich-Edwards et al compared the impact of web-based lifestyle intervention Heart Health 4 Moms (HH4M) vs. general online information (control group) on CVD risk factors in American women (*n* = 151) with a history of preeclampsia [38]. At nine months, women in the intervention group reported a significant increase in knowledge of CVD risk factors (*p* = 0.01) and self-efficacy for health eating (*p* = 0.03) and a reduction in sedentary behavior (*p* = 0.0006), but found no differences in physical activity levels, DASH diet score, blood pressure of weight between groups [38]. Notably, the current study also demonstrated a moderate effect size for physical activity (−0.57), however results favored the control group. This is potentially due to the self-report tool, which may be insensitive to the changes in physical activity promoted by the intervention and likely to be made by postpartum women. Furthermore, the study had high rates of implausible data with the tools (*n* = 24/31 at baseline, 18/24 at follow-up). Therefore, future trials should consider the use of an objective assessment of physical activity, such as accelerometers, to better assess this outcome. This is consistent with the findings and conclusions of Rich-Edwards et al [38] who, despite demonstrating significant changes in physical inactivity, were unable to detect any subsequent changes in physical activity levels.

Overall, the preliminary efficacy findings highlight a challenge faced by CVD prevention research targeting women following preeclampsia or other pregnancy complications associated with increased CVD risk. The sample recruited were up to four years postpartum, and despite having a higher risk of CVD due to their pregnancy history, very few participants entered the study with other CVD risk factors, such as hypertension or hypercholesterolemia, and most were considered low risk of CVD according to the Framingham 30-year CVD Risk Score. The only prevalent CVD risk factor was overweight and obesity. There is evidence to suggest that following preeclampsia, increased rates of cardiovascular events are observed within 10 years [10,11], therefore providing lifestyle intervention following pregnancy is optimal. Therefore, it is imperative that future trials evaluating the efficacy of CVD prevention interventions among women following preeclampsia recruit an appropriately powered sample, and have sufficient long-term follow-up to allow the impact on key CVD risk factors (e.g., blood pressure) to be detected in this younger cohort. Further, focusing on changes to other CVD risk factors, such as weight, diet, physical activity and stress, as a primary outcome, may be favorable due to potential for shorter-term impact. 

### 4.4. Study Strengths and Limitations

The strengths of this pilot study include the RCT study design and the new intervention approach to CVD prevention based on formative research in this under-served target group. The acceptability of the intervention approach was robustly assessed, although there were limitations to the scope of the objectively measured intervention usage data available (e.g., no data on number of logins or page visits). Due to the small sample size and short-term follow-up the results to estimate the impact of the intervention on CVD risk, risk markers, health behaviors and health and well-being are inconclusive. 

## 5. Conclusions

To our knowledge this is only the second RCT among women following preeclampsia to evaluate an intervention to prevent CVD. The results of this RCT demonstrate the potential of a web-based lifestyle behavioral intervention targeting women with a recent history of preeclampsia. Participant acceptability data will be used to inform future iterations of the Be Health*e* for your Heart program, with changes to the existing web-based program, along with either additional technology or in-person delivery of intervention content to be further investigated. Our formative research [22], along with the current study’s recruitment rates and acceptability data provide strong evidence to demonstrate that women with a history of preeclampsia are interested in web-based lifestyle interventions for CVD prevention. Therefore, further research is warranted to continue to inform the implementation of effective CVD prevention interventions in this group.

## Figures and Tables

**Table 1:** Participant inclusion and exclusion criteria.

Inclusion Criteria	Exclusion Criteria
History of preeclampsia (within four years of diagnosis)	Currently or recently pregnant (<3 months postpartum)
Aged 18 to 45 years	Planning to become pregnant within the next three months
Internet access and email address	Non-English speaking
Able to attend assessments at The University of Newcastle Callaghan campus	Type I or II Diabetes Mellitus due to potential impact on secondary outcomes
Interested in all or some of the topics below: (a) Improving eating habits. (b) Improving physical activity levels (c) Managing their body weight (d) Managing their stress	Currently participating in another lifestyle behavior intervention
Completed postpartum check-up at six weeks with no further follow-up required	Unable to provide the contact details of their General Practitioner to allow for follow-up pf any identified concerns from measurement of cardiovascular risk markers

**Table 2:** Preliminary efficacy secondary outcomes measured in the Be Health*e* for your Heart pilot randomized controlled trial ^1^.

Outcome	Description
**Absolute CVD Risk**	
Absolute full CVD risk	Determined using the *Framingham CVD 30-year risk score* [28]. The score considers age, sex, total and high-density lipoprotein cholesterol (HDL-C), current smoking status, systolic blood pressure, use of antihypertensive treatment, and diagnosis of diabetes
**CVD Risk Markers**	
Weight	Measured to the nearest 0.01 kg on a digital scale
Body mass index (BMI)	Calculated from measured height and weight using the standard equation: weight (kg)/height (m^2^)
Waist circumference	Measured to the nearest 0.1 cm using a non-extensible steel tape measure
Blood pressure	Systolic and diastolic blood pressure were measured using an automatic sphygmomanometer
Cardiovascular biomarkers	Fasted blood samples were collected to measure total cholesterol, high-density lipoprotein (HDL-C), low-density lipoprotein cholesterol (LDL-C), triglycerides, glucose and insulin
**Health Behaviors**	
Physical activity	The International Physical Activity Questionnaire (IPAQ) (short-form) was used to calculate MET-minutes per week and categorize physical activity level (low, medium or high) [29]. Participation in resistance-based physical activity was also assessed (duration and frequency).
Sitting time	The Domain-Specific Sitting Questionnaire (adapted version) was used to assess weekday and weekend-day sitting time across five domains [30,31]
Dietary intake	The Australian Eating Survey Food Frequency Questionnaire (AES FFQ)—CVD version was used to assess dietary intake, including 66 supplementary questions specific to foods and nutrients related to CVD health [32]
Depression, anxiety and stress	The Depression, Anxiety and Stress Scale (DASS) (long version) was used to assess depression, anxiety and stress on individual scales [33] ^2^
**General Health and Wellbeing**	
Quality of life	The Quality of Life Enjoyment and Satisfaction Questionnaire Short Form (Q-LES-Q-SF) [34] and Satisfaction with Life Scale (SWLS) were used to assess quality of life and satisfaction with life [35]

^1^ Detailed description of measurement of secondary outcomes provided in protocol [21]. ^2^ Long version of DASS unintentionally included in survey instead of short version as indicated in original protocol [21].

**Table 3:** Baseline characteristics of women (*n* = 31) participating in the Be Health*e* for your Heart three-month lifestyle behavior intervention for women with a history of preeclampsia randomized controlled trial.

Characteristic	Total (*n* = 31)	Intervention (*n* = 16)	Control (*n* = 15)
**Socio-Demographic Characteristics**			
**Age (years)**	33.4 ± 4.6	33.6 ± 4.6	33.1 ± 5.1
**Country of birth**			
Australia	93.6 (29)	93.8 (15)	93.3 (14)
Other	6.4 (2)	6.3 (1)	6.7 (1)
**Marital status**			
Never married	16.1 (5)	18.8 (3)	13.3 (2)
Married	80.7 (25)	75.0 (12)	86.7 (13)
Separated/divorced	3.2 (1)	6.3 (1)	0 (0)
**Highest education level completed**			
University degree	48.4 (15)	43.8 (7)	53.3 (8)
Diploma or Trade	35.5 (11)	15.1 (4)	46.7 (7)
High school	16.2 (5)	14.3 (5)	0
**Combined household income**			
≥$2000/week	32.2 (10)	37.5 (6)	26.7 (4)
≥$1000/week and <$2000/week	45.2 (14)	56.3 (9)	33.3 (5)
<$1000/week	3.2 (1)	0	6.7 (1)
Do not know or wish to answer	5	0	33.3 (5)
**Number of children/dependents**			
Total	1.9 ± 0.9	1.9 ± 1.1	1.9 ± 0.7
Under 2 years	0.6 ± 0.6	0.6 ± 0.5	0.6 ± 0.6
2 to 5 years	1.0 ± 0.8	0.9 ± 0.7	1.1 ± 0.8
6 to 10 years	0.2 ± 0.4	0.2 ± 0.4	0.1 ± 0.4
11 years and over	0.1 ± 0.5	0.2 ± 0.8	0 ± 0
**Pregnancy and preeclampsia history**			
Number of pregnancies	2.6 ± 2.0	3.1 ± 2.5	2.1 ± 1.4
Number of births	1.7 ± 0.7	1.8 ± 0.6	1.6 ± 0.9
**Number of pregnancies complicated by preeclampsia**			
One	80.7 (25)	81.3 (13)	80.0 (12)
Two	19.4 (6)	18.8 (3)	20.0 (3)
**Time since most recent pregnancy complicated by preeclampsia**			
3 months to <1 year	22.6 (7)	18.8 (3)	26.7 (4)
≥1 to <2 years	9.7 (3)	6.3 (1)	13.3 (2)
≥2 to 4 years	67.7 (21)	75.0 (12)	60.0 (9)
**Time of preeclampsia diagnosis ^a^**			
< 34 weeks gestation	60.0 (18)	73.3 (11)	46.7 (7)
34–37 weeks gestation	10.0 (3)	6.7 (1)	13.3 (2)
≥ 37 weeks gestation	20.0 (6)	20.0 (3)	20.0 (3)
Postpartum gestation	10.0 (3)	0	20.0 (3)
**Pregnancy outcome**			
Live birth (> 37 weeks)	51.6 (16)	56.3 (9)	46.7 (7)
Live preterm birth (< 37 weeks)	45.2 (14)	37.5 (6)	53.3 (8)
Stillbirth	3.2 (1)	6.3 (1)	0
**Absolute CVD risk**			
**Framingham CVD 30-year Risk (Hard) (%)**	7.2 ± 4.2	5.8 ± 3.4	8.7 ± 4.6
Low risk (<10%)	80.0 (24)	86.7 (13)	73.3 (11)
Intermediate risk (10–20%)	20.0 (6)	13.3 (2)	26.7 (4)
High risk (>20%)	0	0	0
**CVD Risk Markers**			
**Weight (kg)**	80.3 ± 20.6	72.6 ± 11.7	88.5 ± 25.0
**Body mass index (kg/m^2^)**	29.8 ± 7.3	27.1 ± 4.4	32.7 ± 8.7
Healthy (18.5 to 24.9)	19.4 (6)	37.5 (6)	0
Overweight (25.0 to 29.9)	45.2 (14)	43.8 (7)	46.7 (7)
Obese (≥ 30.0)	35.5 (11)	18.8 (3)	53.3 (8)
**Waist circumference (cm)**	91.9 ± 13.7	87.1 ± 9.1	96.9 ± 16.2
**Body fat (%)**	38.6 ± 8.2	35.9 ± 7.6	41.5 ± 7.9
**Blood pressure (mmHg)**			
Systolic	111.6 ± 13.9	104.9 ± 10.5	118.6 ± 13.8
Diastolic	76.0 ± 8.5	73.4 ± 7.1	78.7 ± 9.2
**Cardiovascular blood biomarkers ^a^:**			
**Total cholesterol (mmol/L)**	4.7 ± 0.9	4.5 ± 1.1	4.8 ± 0.8
**HDL-C (mmol/L)**	1.4 ± 0.3	1.5 ± 0.3	1.2 ± 0.3
**LDL-C (mmol/L)**	2.8 ± 0.8	2.6 ± 1.0	3.0 ± 0.6
**Triglycerides (mmol/L)**	1.0 ± 0.6	0.8 ± 0.6	1.2 ± 0.6
**Glucose (mmol/L)**	4.7 ± 0.6	4.5 ± 0.6	4.8 ± 0.6
**Insulin (mIU/L)**	10.0 ± 8.5	6.5 ± 3.3	13.4 ± 10.7
**Health Behaviours**			
**Physical activity (MET min/week) ^b^:**	2304 ± 2497	2345 ± 2720	2256 ± 2337
**Resistance-based activities (minutes/week)**	31±63	28±58	33±70
**Sitting time**			
Weekdays (minutes/day)	495 ± 214	423 ± 176	571 ± 230
Weekend days (minutes/day)	480 ± 161	473 ± 181	488 ± 142
**Dietary intake**			
Total energy (kJ/day) (kcal/day)	9097 ± 2852 2174 ± 682	9712 ± 2414 2321 ± 577	8441 ± 3207 2018 ± 767
Discretionary energy (kJ/day) (kcal/day)	3132 ± 1487 749 ± 355	3318 ± 1443 793 ± 345	2934 ± 1558 701 ± 372
Discretionary (% energy)	34.8 ± 12.2	34.3 ± 11.9	35.3 ± 13.0
Protein (% energy)	18.9 ± 3.8	18.8 ± 3.5	19.1 ± 4.3
Fats (% energy)	37.7 ± 5.6	37.9 ± 6.3	37.6 ± 5.1
Saturated fat (% energy)	13.4 ± 2.7	13.2 ± 2.4	13.6 ± 3.0
Monounsaturated fat (% energy)	15.3 ± 2.9	15.6 ± 3.0	14.9 ± 2.9
Polyunsaturated fat (% energy)	5.9 ± 1.6	5.9 ± 1.6	6.0 ± 1.6
Fibre (g)	28.7 ± 11.8	30.8 ± 10.9	26.4 ± 12.6
Sodium (mg)	2034 ±586	2165 ± 546	1904 ± 615
Fruit (serves/day)	1.3 ± 1.0	1.4 ± 1.1	1.3 ± 1.0
Vegetable (serves/day)	4.2 ± 2.0	4.6 ± 2.1	3.9 ± 1.8
Nuts (serves/day)	0.4 ± 0.5	0.5 ± 0.5	0.4 ± 0.5
Fish (serves/day)	0.2 ± 0.2	0.2 ± 0.2	0.2 ± 0.2
Legumes (serves/day)	0.4 ± 0.7	0.4 ± 0.6	0.4 ± 0.8
**Depression, Anxiety and Stress Scale**			
Depression Score (0–42 points)	4.7 ± 4.5	4.0 ± 3.5	5.4 ± 5.5
Anxiety Score (0–42 points)	6.3 ± 5.6	5.6 ± 4.8	7.1 ± 6.5
Stress Score (0–42 points)	8.5 ± 5.7	7.9 ± 4.4	9.1 ± 7.0
**General Health and Well-Being**			
**Quality of Life**			
Q-LES-Q-SF Score (%)	59.9 ± 16.0	58.5 ± 14.9	61.3 ± 17.5
**Satisfaction with Life Scale**			
Overall score (Max: 35 points)	25.8 ± 5.2	25.9 ± 3.8	25.7 ± 6.6
Extremely satisfied (31–35 points)	12.9 (4)	6.3 (1)	20.0 (3)
Satisfied (26–30 points)	51.6 (16)	62.5 (10)	40.0 (6)
Slightly satisfied (21–25 points)	19.4 (6)	18.8 (3)	20.0 (3)
Neutral (20 points)	6.5 (2)	6.3 (1)	6.7 (1)
Slightly dissatisfied (15–19 points)	3.2 (1)	6.3 (1)	0
Dissatisfied (10–14 points)	6.5 (2)	0	13.3 (2)

Data are presented as mean ± SD or % (n). ^a^ Data available for 30 participants for Number of births, Gestation at time of preeclampsia diagnosis, Cardiovascular blood measures, and Framingham risk score. ^b^ Data available for 24 participants for Physical activity. HDL-C, High-density lipoprotein cholesterol; LDL-C, Low-density lipoprotein cholesterol; Q-LES-Q-SF, The Quality of Life Enjoyment and Satisfaction Questionnaire—Short Form; Max, maximum possible score for the scale, Met, Metabolic Equivalents; SD, Standard deviation.

**Table 4:** Mean ± SD acceptability of the Be Health*e* for your Heart program components among intervention participants.

Program Components	How Healthy is Your Heart? (*n* = 11)	My Goals (*n* = 7)	Track My Progress (*n* = 4)	Website Resources (*n* = 12)	Email Newsletters (*n* = 10)
Useful information about healthy eating	4.4 ± 0.5	NA	4.3 ± 0.4	4.3 ± 0.4	4.2 ± 0.4
Useful information about exercise	4.3 ± 0.4	NA	4.3 ± 0.4	4.1 ± 0.3	4.2 ± 0.4
Useful information about weight management	4.1 ± 0.5	NA	3.8 ± 0.8	4.1 ± 0.5	4.2 ± 0.4
Useful information about stress management	4.1 ± 0.5	NA	4.3 ± 0.4	4.1 ± 0.5	4.2 ± 0.4
Helped me to attain my goals	3.6 ± 0.9	3.4 ± 0.5	4.0 ± 0.7	3.6 ± 0.8	3.8 ± 0.7
Motivated me	4.0 ± 0.7	3.9 ± 0.6	4.0 ± 1.2	3.8 ± 0.6	4.1 ± 0.7
Made me feel accountable	4.1 ± 0.9	3.9 ± 0.6	4.3 ± 0.8	3.8 ± 0.6	4.2 ± 0.6
Was easy to access/use	4.2 ± 0.4	4 ± 0.0	4.3 ± 0.4	4.0 ± 0.4	4.2 ± 0.4
Was visually appealing	4.4 ± 0.5	NA	NA	4.1 ± 0.3	4.2 ± 0.4
**Overall Component satisfaction (*n* = 13)**	**4.2 ± 0.4**	**3.7 ± 0.4**	**3.6 ± 0.8**	**4.0 ± 0.6**	**4.2 ± 0.7**

**Table 5:** Changes in secondary outcome measures from baseline to three months for women (*n* = 31) participating in the Be Health*e* for your Heart three-month lifestyle behavior intervention for women with a history of preeclampsia randomized controlled trial.

Outcome Measures	Mean (95% CI) Change from Baseline to 3 Months		Mean Difference between Groups	Effect Size (Cohens *d*)
	Intervention (*n* = 16)	Control (*n* = 15)		
**Absolute CVD Risk**				
**Framingham CVD-30 years Risk Score**	0.4 (−0.5, 1.3)	0.9 (−0.1, 1.9)	−0.5 (−0.9, 1.9)	−0.12
**CVD Risk Markers**				
**Weight (kg)**	−0.1 (−1.5, 1.3)	−0.1 (−1.6, 1.4)	0.1 (−2.1, 2.0)	0.00
**Body mass index (kg/m^2^)**	−0.04 (−0.6, 0.5)	−0.1 (−0.7, 0.5)	0.1 (−0.8, 0.7)	0.00
**Waist circumference (cm)**	−0.7 (−3.0, 1.7)	−0.6 (−3.2, 1.9)	−0.1 (−3.4, 3.5)	−0.00
**Body fat (%)**	0.9 (−1.3, 3.1)	0.4 (−2.0, 2.8)	−0.5 (−3.7, 2.8)	−0.06
**Blood pressure (mmHg)**				
Systolic	3.2 (−1.6, 8.0)	−1.0 (−6.2, 4.1)	4.2 (−11.3, 2.8)	0.30
Diastolic	3.1 (−1.1, 7.3)	1.2 (−3.3, 5.7)	1.9 (−8.1, 4.3)	0.23
**CVD biomarkers**				
**Total chol. (mmol/L)**	0.01 (−0.5, 0.5)	0.5 (0.04, 1.04) *	−0.5 (−0.2, 1.2)	−0.58
**HDL-C (mmol/L)**	−0.0001 (−0.2, 0.1)	0.1 (−0.1, 0.2)	−0.1 (−0.1, 0.3)	−0.27
**LDL-C (mmol/L)**	−0.1 (−0.5, 0.4)	0.4 (−0.1, 0.8)	−0.5 (−0.2, 1.1)	−0.56
**Triglycerides (mmol/L)**	0.1 (−0.1, 0.4)	0.1 (−0.2, 0.4)	0.002 (−0.4, 0.4)	0.00
**Glucose (mmol/L)**	0.03 (−0.3, 0.4)	−0.3 (−0.7, 0.03)	0.4 (-0.9, 0.1)	0.60
**Insulin (mIU/L)**	1.0 (−1.8, 3.6)	−2.5 (−5.4, 0.4)	3.4 (−7.4, 0.5)	0.40
**Health Behaviours**				
**Physical activity**				
**Physical activity (MET min/week)**	−863 (−1965, 239)	551 (−829, 1930)	−1413 (−354, 3181)	−0.57
**Resistance training (min/week)**	25 (−7,57)	−21 (−56,14)	−47 (−94, 1)	−0.75
**Sitting time**				
Weekdays (min/day)	−32 (−128, 65)	−1 (−105, 103)	−30 (−112, 173)	−0.14
Weekend days (min/day)	−53 (−133, 27)	−45 (−131, 41)	−8 (−109, 125)	−0.05
Dietary intake				
Total energy (kJ/day) (Kcal/day)	−466 (−1555, 622) −111 (−372,149)	97 (−1080, 1274) 23 (−258,305)	−563 (−1041, 2167) −135 (−249,518)	−0.20
Discretionary (% energy)	−0.03 (−4.5, 4.4)	0.8 (−4.1, 5.6)	−0. 8 (−5. 8, 7.3)	−0.06
Protein (% energy)	0.2 (−1.8, 2.2)	−0.1 (−2.2, 2.1)	0.3 (−3.2, 2.7)	0.08
Fats (% energy)	−0.4 (−2.1, 1.2)	−2.5 (−4.3, −0.7)	2.1 (−4.5, 0.3)	0.37
Saturated fat (% energy)	−0.01 (−0.9, 0.9)	−1.2 (−2.2, -0.2)	1.2 (−2.5, 0.2)	0.43
MUFA (% energy)	−1.0 (−2.1, 0.1)	−0.6 (−1.8, 0.6)	0.4 (−1.3, 2.0)	0.13
PUFA (% energy)	0.3 (−0.6, 1.3)	−0.5 (−1.5, 0.5)	0.8 (−2.2, 0.6)	0.51
Fibre (g)	0.3 (−3.9, 4.4)	1.2 (−3.2, 5.7)	−1.0 (−5.1, 7.0)	−0.08
Sodium (mg)	−94 (−361, 173)	267 (−22, 556)	−361 (−32, 754)	−0.62
Fruit (serves/day)	0.4 (−0.03, 0.7)	0.3 (−0.1, 0.7)	0.1 (−0.6, 0.5)	0.07
Vegetable (serves/day)	0.4 (−0.5, 1.2)	−0.2 (−1.2, 0.7)	0.6 (−1.9, 0.7)	0.31
Nuts (serves/day)	−0.3 (−0.5, 0.01)	−0.1 (−0.3, 0.2)	−0.2 (−0.2, 0.6)	−0.38
Fish (serves/day)	0.1 (−0.01, 0.1)	−0.01 (−0.1, 0.1)	0.1 (−0.1, 0.03)	0.30
Legumes (serves/day)	0.1 (−0.03, 0.1)	0.1 (−0.03, 0.2)	−0.01 (−0.1, 0.1)	−0.01
**Depression, Anxiety and Stress Scale**				
Depression	−0.3 (−2.4, 1.8)	−1.7 (−4.0, 0.5)	1.5 (−4.5, 1.6)	0.32
Anxiety	−0.5 (−3.2, 2.3)	−2.4 (−5.3, 0.6)	1.9 (−5.9, 2.1)	0.34
Stress	−0.9 (−3.6, 1.7)	−2.2 (−5.1, 0.7)	1.2 (−5.2, 2.7)	0.22
**General health and wellbeing**				
Q-LES-Q-SF Score (%)	5.7 (−1.9, 13.2)	4.5 (−3.7, 12.6)	1.2 (−12.3, 9.9)	0.08
Satisfaction with Life Scale overall score	0.6 (−1.8, 2.9)	1.1 (−1.5, 3.6)	−0.5 (−3.0, 4.0)	−0.10

* *p* < 0.05; all other results *p* > 0.05. Chol, Cholesterol; HDL-C, High-density lipoprotein cholesterol; LDL-C, Low-density lipoprotein cholesterol; MET, Metabolic Equivalent; MUFA, monounsaturated fat; PUFA, polyunsaturated fat; Q-LES-Q-SF, The Quality of Life Enjoyment and Satisfaction Questionnaire—Short Form score.
